# A Redactable Blockchain-Based Data Management Scheme for Agricultural Product Traceability

**DOI:** 10.3390/s24051667

**Published:** 2024-03-04

**Authors:** Shangxiong Yang, Shaowen Li, Wenjia Chen, Yawen Zhao

**Affiliations:** 1 School of Information and Artificial Intelligence, Anhui Agricultural University, Hefei 230036, China; 2 Key Laboratory of Agricultural Sensors, Ministry of Agriculture and Rural Affairs, Hefei 230036, China

**Keywords:** agricultural product traceability, redactable blockchain, chameleon hash, attribute-based encryption, data management

## Abstract

With the development of agricultural information technology, the Internet of Things and blockchain have become important in the traceability of agricultural products. Sensors collect real-time data in agricultural production and a blockchain provides a secure and transparent storage medium for these data, which improves the transparency and credibility of agricultural product traceability. However, existing agricultural product traceability solutions are limited by the immutability of the blockchain, making it difficult to delete erroneous data and modify the scope of data sharing. This damages the credibility of traceability data and is not conducive to the exchange and sharing of information among enterprises. In this article, we propose an agricultural product traceability data management scheme based on a redactable blockchain. This scheme allows agricultural enterprises to encrypt data to protect privacy. In order to facilitate the maintenance and sharing of data, we introduce a chameleon hash function to provide data modification capabilities. Enterprises can fix erroneous data and update the access permissions of the data. To improve the efficiency of block editing, our scheme adopts a distributed block editing method. This method supports threshold editing operations, avoiding single-point-of-failure issues. We save records of data modifications on the blockchain and establish accountability mechanisms to identify malicious entities. Finally, in this paper we provide a security analysis of our proposed solution and verify its effectiveness through experiments. Compared with the existing scheme, the block generating speed is improved by 42% and the block editing speed is improved by 29.3% at 125 nodes.

## 1. Introduction

With the rapid development of technology, daily life has become more convenient and our quality of life has been significantly improved. However, frequent food and agricultural product safety incidents in recent years have posed a serious threat to consumers’ physical health and the reputation of numerous enterprises [1,2,3]. These security incidents not only pose potential risks to individuals but also negatively impact businesses. In this current situation, ensuring the safety and traceability of agricultural products has become an important issue that urgently needs to be addressed. Through full process information recording, agricultural product traceability [4] ensures transparency in product sources, production conditions, processing processes, and other aspects. Enterprises and individuals can query and conduct a statistical analysis of this information to gain a more comprehensive understanding and supervision of the production process and the circulation of agricultural products. Such comprehensive information acquisition and supervision helps to improve the overall quality of agricultural products and provides consumers with more reliable products [5].

However, traditional traceability systems often use a single central database [6] and traceability data are easily tampered with, allowing for criminals to forge information and conceal violations [7]. Traditional traceability systems have numerous shortcomings, such as slow information flow and insufficient transparency [8], making it difficult for consumers and regulatory agencies to obtain real-time and accurate information, which hinders the use of the system. These issues constrain the practicality of traceability systems in modern agricultural environments. Therefore, various researchers [9,10,11] have proposed the use of blockchain-based agricultural product traceability. Blockchain [12] is a distributed ledger technology that records transactions and information through a series of inter-connected blocks. Blockchains maintain and verify data through entities in the network and are highly stable and secure [13]. Entities in the network are free to view the data in the blockchain. Consumers and businesses have access to secure and stable services for data queries [14].

However, their immutability also poses challenges for applying blockchain in agricultural product traceability. Traditional blockchains require each entity to store complete blockchain data. With the continuous growth of blockchains, the amount of data is also constantly expanding, which may lead to an increase in the storage and processing burden on the entities [15]. Moreover, there may be illegal content on the blockchain which could violate the relevant laws [16]. Malicious attacks on the blockchain may also result in incorrect information being uploaded [17]. In terms of this, Ateniese et al. [18] proposed a redactable blockchain that replaces SHA-256 with a chameleon hash function [19], allowing entities holding trapdoor keys to modify the blockchain. Studies have been conducted on the use of the redactable blockchain in the industrial Internet of Things [20,21,22], smart grids [23,24], and medical fields [25,26], except for agricultural product traceability. The failure information in the blockchain-based agricultural product traceability system is difficult to modify and there is a gap in promoting redactable blockchains in agricultural product traceability.

On the other hand, existing agricultural product traceability systems typically use attribute-based encryption algorithms [27] to control data access permissions to protect the privacy and trade secrets of various enterprises [28,29,30]. In traditional blockchain-based agricultural product data management systems, due to the immutability of blockchains, data access permissions cannot be changed once uploaded, which is not conducive to the flexible control of access permissions. Therefore, existing solutions suffer from the problem that data access policies cannot be changed. Introducing a redactable blockchain can lift this restriction and reasonably modify the access control permissions for the data on the blockchain.

In recent years, many redactable blockchain solutions have been proposed [18,22,31,32,33]; these can be divided into centralized and decentralized solutions based on the holding of the editing permissions. In centralized solutions [18,32,33], a trusted third-party entity holds the trapdoor key and rewrites the block, which results in centralized rights, meaning that it is not suitable for distributed application scenarios. In decentralized solutions [22,31], the trapdoor key is split into multiple entities and, when modifying blocks, multiple entities need to cooperate to complete it. These methods usually require the participation of all entities when rewriting blocks, resulting in a low editing efficiency and many of them do not provide accountability mechanisms to find malicious entities. However, these decentralized redactable blockchain solutions require the participation of all entities when rewriting blocks, resulting in a slow execution speed. Moreover, the editing process of the existing schemes is vulnerable to malicious entities. Therefore, existing solutions are problematic, with low editing efficiency and a lack of accountability mechanisms to identify malicious entities.

In response to the above issues, we propose an agricultural product traceability data management scheme based on the redactable blockchain. In contrast to previous solutions, our proposal explores the application of redactable blockchains in agricultural product traceability. Furthermore, our approach integrates the requirement of safeguarding privacy with the necessity of sharing data. Our solution uses decentralized storage to ensure that there is no single point of failure. From a technical standpoint, our solution not only provides support for data access control but also introduces the modification of erroneous data and access control policy updates. This aspect has not been thoroughly investigated in previous agricultural blockchain traceability scenarios. Our solution divides the decision-making authority for data maintenance among authorized entities, ensuring that the system operations are efficient. In order to secure the data, any modifications to the data are recorded on the blockchain, which can provide a source material for audits by government regulators. The main contributions of this study are as follows:

- We design a storage architecture based on the redactable blockchain for agricultural product traceability data for the first time. By using a redactable blockchain, the architecture achieves the distributed storage of data. The architecture uses cryptographic algorithms to encrypt data and control data access permissions, to protect enterprise privacy. The re-encryption algorithm provides the necessary conditions for updating access permissions. The architecture combines a method of storing raw data off-chain and data summaries on-chain to alleviate the storage pressure on the blockchain entities.
- We provide maintenance methods for on-chain data by introducing the redactable blockchain. Enterprises can modify erroneous data and update the access permissions of the data. This solves the problem of outdated data being difficult to update and enhances the credibility and shareability of the agricultural product traceability data.
- We propose a novel distributed redactable blockchain solution by improving the chameleon hash function. Authorized entities that reach the threshold can edit blocks, reduce the impact of malicious entities, and improve the efficiency of on-chain data maintenance. In addition, we propose an accountability mechanism to identify malicious entities attempting to obstruct the block editing, ensuring the security of the system.

The remainder of this paper is organized as follows: In Section 2, we introduce related work on agricultural product traceability and the redactable blockchain. In Section 3, we introduce the background of our research. In Section 4, we present an overview of our proposed scheme. In Section 5, we provide the implementation details of our scheme. In Section 6, we perform a security analysis. In Section 7, we test the performance of our scheme. Finally, in Section 8, we provide the conclusions of our study.

## 2. Related Work

### 2.1. Blockchain-Based Agricultural Traceability Systems

Traceability [34] refers to the ability to trace the entire process of product production, processing, transportation, etc., to ensure that the source and various stages of the product can be traced at any time. By establishing a systematic traceability system, detailed information such as the production location, growth conditions, picking time, processing process, and transportation route of agricultural products can be traced [35]. This enables consumers, producers, and regulatory agencies to obtain comprehensive information about products, thereby enhancing their confidence in product quality, safety, and compliance. Traceability is considered an important quality management tool in the agricultural field, which helps to improve product transparency, strengthen regulation, and enhance the efficiency of the entire supply chain [11].

In the field of agriculture, researchers are committed to developing or introducing new technologies to enhance the security and reliability of agricultural product supply, including Quick Response (QR) Codes [36], P-fertilizer supply chain management (PFSCM) [37], Radio Frequency Identification (RFID) [38], the Internet of Things (IoT) [39], and blockchain technology [40].

Blockchain, through its distributed ledger technology, ensures the transparent sharing of information in the network and enhances the credibility and stability of agricultural product traceability systems. Studies [7,8,9,10,11,28,29,30,41,42,43,44] have been performed where a blockchain is applied to product traceability. Ibtisam et al. [41] proposed a fully decentralized traceability model based on blockchain, which provides product source verification, product transportation monitoring, and transaction documentation to ensure system integrity and transparency. The model also uses smart contracts to achieve efficient and traceable agricultural product transactions and consumers can call smart contracts to view the entire production and processing process information of agricultural products. Francisco et al. [42] deployed an embedded device network using IoT protocols and control algorithms, creating a consortium blockchain that provides tamper-proof, transparent, and secure traceability services. However, these studies often failed to consider the handling of erroneous data and were unable to effectively update on-chain information.

The agricultural product traceability system contains information related to production, transportation, processing, etc., which may include sensitive information such as commercial secrets and personal identity information within the enterprise [43]. Therefore, some studies [28,29,30,44] have incorporated access control functions in the system. In Hyperledger Fabric [44], access control is implemented through identity authentication, authorization policies, and channel configuration to ensure that the participants in the network can only perform operations that they have permission to. This approach is not conducive to the transparency of data and is also difficult to achieve complex access control. Therefore, many people use attribute-based encryption algorithms to implement access control in traceability systems.

Zhang et al. [28] integrated blockchain technology with the CP-ABE algorithm and applied it to a secure and trustworthy agricultural product traceability system. This system designed an attribute management infrastructure that can monitor and efficiently manage the attributes of the entire blockchain. Xu et al. [29] used Internet of Things technology to collect fish source and growth data, and they also used attribute-based encryption to achieve data access control and stored the data in the blockchain to achieve the reliable storage of fish farming data.

Lv et al. [14] reviewed the research on the blockchain-based traceability of agricultural products in the past decade (2013–2023), summarized the core issues and technologies, and introduced the advantages and disadvantages of blockchain. They pointed out that optimizing existing blockchain or combining it with emerging technologies is beneficial for developing agricultural product traceability.

In agricultural product traceability systems, there may be situations where access control rules need to be updated, such as changes in laws and regulations, adjustments to partnership relationships, the emergence of new business models, etc. This may require flexible updates to the access control of the traceability system. The immutability of traditional blockchains limits the flexibility of the system, making it unable to respond to new access control requirements promptly. Therefore, introducing a redactable blockchain into blockchain-based traceability systems is meaningful.

### 2.2. Data Management Schemes Based on Redactable Blockchains

Deuber et al. [31] proposed a CV-chain that extends the block header structure to record old states, maintains connectivity between the edited block and the next block, and uses voting to reach a consensus on editing. The drawback of this scheme is its low editing efficiency. The frequent editing of blocks will lead to longer consensus delays and even more forks. In addition, this scheme has poor compatibility with typical blockchain protocols.

Ateniese et al. [18] first used the chameleon hash function to replace the SHA-256 algorithm and constructed a redactable blockchain. However, their solution only supports block-level editing, which undermines the traceability of normal transactions and relies on multi-party secure computing, resulting in high communication and computing costs. The collision search function requires the explicit restoration of the trapdoor key, which is controlled by a single entity during editing, and there is a risk of arbitrarily modifying blocks.

Derler et al. [32] proposed a policy-based chameleon hash function, which controls editing permissions with access policies. This scheme provides transaction-level editing and fine-grained access control, but this scheme does not implement accountability, and entities that meet the permissions can freely modify blocks. Tian et al. [33] proposed and implemented a redactable blockchain that supports anonymous editing and accountability. Entities that meet permissions can anonymously modify the blockchain, and the uploader of the original transaction can hold the malicious alterer accountable. To manage keys, their scheme requires complex cryptographic operations, which will result in lower computational efficiency. Furthermore, Duan et al. [45] proposed a strongly accountable scheme that can hold accountable the attacker of access permissions. These schemes that rely on attribute-based encryption do not often consider updating access policies and, more importantly, the concentration of editing or accountability rights may lead to single-point-of-failure issues, making them unsuitable for distributed environments.

Huang et al. [22] proposed a threshold chameleon hash (TCH) and a traceable and disinfectable chameleon signature to construct a redactable consortium chain and apply it to the industrial Internet of Things. TCH has implemented distributed collision calculation, but essentially it only provides an n–n threshold. The process of calculating conflicts requires collaborative operation by all sub-key holders, which will increase bandwidth costs and reduce editing efficiency and flexibility. Each edit of the blockchain requires all communications, and if any of the sensors fail, the editing will fail.

We present a comparison in Table 1. Many of these redactable blockchains suffer from centralized editing permissions, where entities with editing rights can freely modify blocks. Others require the participation of all entities when performing modification operations, which leads to lower execution efficiency. And some do not have accountability mechanisms to recognize malicious entities.

In other fields, some studies [20,21,22,23,24,25,26,46,47,48,49,50] have applied redactable blockchains to data management and sharing. He et al. [25] proposed a shared medical data management scheme based on a redactable blockchain, which uses a chameleon hash function to construct a redactable blockchain and encrypt medical data. Private data sharing is negotiated between patients and medical institutions. The disadvantage of this scheme is that the trapdoor key needs to be explicitly restored before editing the block, which poses a risk of key exposure. Wei et al. [20] proposed a redactable blockchain framework for secure federated learning in the industrial Internet of Things, which uses sensor devices to collect data for federated learning and utilizes editability to delete private and erroneous data. Xu et al. [46] applied redactable blockchain technology to identity management in mobile network environments. They used the decentralized blockchain to improve the trustworthiness of the system. They used redactable blockchain technology to delete unreasonable mobile user information, reduce the storage burden of network operators, and improve the efficiency of identity verification. Yi et al. [47] used blockchain to store digital content to replace third-party organizations and improve the openness and transparency of management. They used perceptual hash to detect the similarity of digital content. When plagiarized or illegal content was found, they used redactable blockchain technology to remove this content. Yang et al. [23] utilized CP-ABE technology to manage access to private data in the smart grid. They used redactable blockchain technology to store energy transactions and to provide users with the ability to modify private data. There is also research on using redactable blockchains to store sensor data in IoT applications [20,21,22,24,50]. At present, there is a lack of research on redactable blockchains in agricultural product traceability.

## 3. Preliminaries

This section introduces various technologies related to this article, including redactable blockchain technology, the chameleon hash function, and an attribute-based proxy re-encryption algorithm.

### 3.1. Redactable Blockchain

Blockchain is a distributed ledger technology used to record transactions or data, which are stored in the form of blocks and connected into a chain in chronological order. Each block contains a batch of transactions or data records and the blockchain is a continuous concatenation of these blocks. Once data are added to the blockchain, it is difficult to modify or delete these data. For most blockchain technologies, miners collect transactions and package a batch of transactions into a block. Miners run a consensus algorithm [51] to determine whether to add a block to the blockchain.

Redactable blockchains are a variant of blockchain technology that allows specific authorized entities to modify or delete specific data records while keeping other data unchanged. This flexibility enables redactable blockchains to be more scalable, while maintaining core traditional blockchain features such as decentralization and immutability.

### 3.2. Chameleon Hash Function

The chameleon hash function [19] is a special hash function originally proposed by Krawczyk and Rabin, which can generate hash collisions under specific conditions. Only those who hold the trapdoor key can find hash collisions and modify the original information without changing the hash value. The basic structure of the chameleon hash function is as follows:

CH.Setup: On inputting security parameters $\lambda_{CH}$, the algorithm outputs system parameters $Param_{CH}$.

CH.KeyGen: On inputting system parameters $Param_{CH}$, the algorithm outputs a public hash key hk and a trapdoor key tk.

CH.Hash: On inputting hash key hk and message m, the algorithm outputs hash value h and randomness r.

CH.Forge: On inputting a new message $m^{\prime }$ and tk, the algorithm outputs a new randomness $r^{\prime }$, satisfying $CH.Hash\left(m,r\right)=Ch.Hash\left(m^{\prime },r^{\prime }\right)$.

### 3.3. Attribute-Based Proxy Re-Encryption

Attribute-based proxy re-encryption (CP-ABPRE) is an encryption technique that combines the characteristics of attribute-based encryption (ABE) and proxy re-encryption. This technology aims to achieve fine-grained access control over encrypted data, allowing data owners to define which users or entities can access their encrypted data and proxy the data to other users when needed. Our scheme adopts the verifiable and fair ciphertext policy attribute-based proxy re-encryption (VF-CP-ABPRE) algorithm proposed by Ge et al. [52], which verifies proxy re-encryption while achieving efficient computational efficiency. The VF-CP-ABPRE scheme consists of the following algorithms:

Setup: Initialize the system. On inputting security parameters $\lambda_{ABE}$, the algorithm outputs system parameters $Param_{ABE}$ and the master secret key msk.

Enc: The data sharer sets access control permissions and encrypts raw information. On inputting message m and access policy AP, the algorithm outputs ciphertext CT.

ReKeyGen: The data sharer sets new access control permissions and generates a re-encryption key. On inputting a private key sk of attribute set S and a new access policy $AP^{\prime }$, the algorithm outputs a re-encryption key rk.

ReEnc: Perform the re-encryption operation. On inputting a re-encryption key rk and ciphertext CT, the algorithm outputs a re-encryption ciphertext $CT^{\prime }$.

OriginDec: A user with access rights can decrypt ciphertext. On inputting a ciphertext CT and a valid secret key sk, the algorithm outputs the origin message m.

ReDec: A user with access rights can decrypt re-ciphertext. On inputting a ciphertext CT, its re-encryption ciphertext $CT^{\prime }$, and a valid secret key sk, the algorithm outputs the origin message m.

Claim: The original uploader of the message can declare the error of re-encryption ciphertext. On inputting a ciphertext CT, its re-encryption ciphertext $CT^{\prime }$, and re-encryption key rk, the algorithm outputs true if $CT^{\prime }$ is incorrect; otherwise, it outputs false.

## 4. System Overview

In this section, we introduce the system model, main stages, threat model, and design goals of the proposed scheme. Table 2 outlines the notations in this paper.

### 4.1. System Model

**Figure 1:**
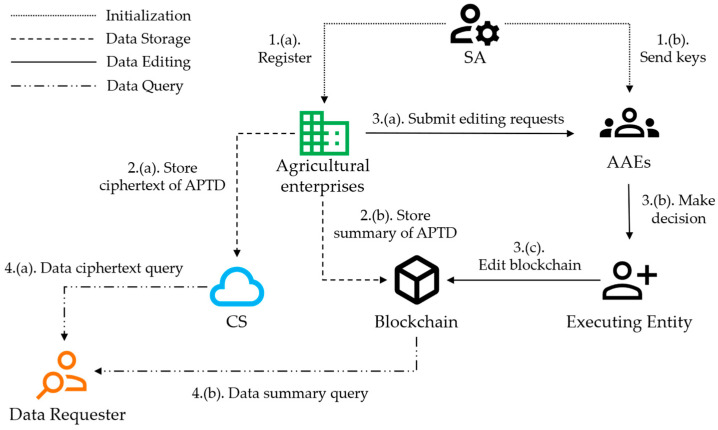
Overview of the agricultural product traceability data management scheme.

To achieve the secure management of agricultural product traceability data (APTD), we propose a redactable blockchain-based APTD management scheme, as shown in Figure 1. The scheme comprises three main entities:

- The system administrator (SA) is responsible for generating the necessary parameters for the system and configuring and launching the blockchain. The SA manages users in the system and is responsible for registering users, assigning user attributes, and distributing keys. In addition, the SA is established and supervised by the relevant government agencies and does not participate in processes such as block creation and block editing.
- Authorized agricultural entities (AAEs) are enterprises engaged in agricultural production and the relevant regulatory agencies. They register with the SA and participate in the traceability of agricultural products. From AAEs, the executing entity can be selected to be responsible for rewriting the blockchain. The rewritten information will only take effect after being uploaded to the blockchain through consensus, so the rights of the executing entity are very limited.
- The cloud server (CS) stores APTD and shares the storage pressure of the blockchain. The CS provides users with APTD query services and provides proxy re-encryption computing services for data sharers. The CS has an extensive storage capacity and efficient computing power.

In addition, the redactable blockchain records APTD’s digital digest and access permissions. Each entity can share and verify the same information on the blockchain. AAEs can edit the block when needed.

### 4.2. Main Stages

The trustworthy agricultural product traceability data management scheme consists of four stages, namely system initialization, data storage, data editing, and data query.

1. System initialization: The SA generates system parameters and initializes the redactable blockchain. Agricultural enterprises and various entities are registered as AAEs with the SA. The SA generates keys and sends them to the AAEs.
2. Data storage: Agricultural enterprises encrypt APTD, store it in the cloud server, and then encrypt the key and upload it to the redactable blockchain. At the same time, the digital digest of the APTD and access policy are also stored on the blockchain.
3. Data editing: Agricultural enterprises or other entities initiate a block edit request to correct erroneous data or update access control permissions for historical data.
4. Data query: Requesters who meet the access rights obtain the ciphertext from the redactable blockchain and CS. Then, they decrypt and view the APTD.

### 4.3. Threat Model

AAEs may be attacked, resulting in the inability to participate in collective computing on time. Malicious entities may refuse reasonable block editing requests. Assuming that there are a sufficient number of non-malicious AAEs in the network, attackers cannot simultaneously attack most AAEs.

Assuming that the consensus mechanism of the blockchain is reliable, the security of the consensus mechanism is not within the scope of this article. Assuming that the SA is able to generate system parameters normally during the initialization phase, in the field of agricultural product traceability, this process should be supervised by regulatory authorities such as the government.

### 4.4. Design Goals

The design goals of our scheme are as follows:

Privacy protection: Agricultural enterprises can effectively modify the access policy of APTD, while malicious entities cannot decrypt and view APTD.

Data security: Decentralize editing permissions. The rewriting of agricultural product traceability data must be approved by the AAEs that exceed the threshold. Without knowing the hash trapdoor key, malicious entities are unable to find hash collisions and maliciously tamper with blocks.

Accountability: The lifecycle of all transactions is honestly recorded in the blockchain and can be effectively audited. The malicious behavior of individual entities does not affect the stability of the system.

## 5. Proposed Scheme

In this section, we first provide an overview of the agricultural product traceability data management scheme based on the redactable blockchain. Subsequently, we introduce the detailed construction of this scheme, which includes four stages, as follows: system initialization stage, data storage stage, data editing stage, and data query stage.

### 5.1. Overview

This article proposes a credible agricultural product traceability data management scheme based on the redactable blockchain. To reduce the storage pressure of the blockchain, we adopt an on-chain and off-chain construction model [53]. Specifically, our scheme uses a symmetric encryption algorithm to encrypt APTD and then uses an attribute-based encryption algorithm to encrypt the key to the symmetric encryption algorithm. Our scheme stores the ciphertext of the data in the CS and uploads the digital digest, the ciphertext of the key, and the access control policy to the redactable blockchain. Our scheme uses the ABPRE algorithm to manage data access rights. Agricultural enterprises define and manage access control policies for their data, which can result in the sharing of traceable data while protecting corporate privacy.

When erroneous data are discovered, the AAE can initiate a block edit to correct the erroneous data. In addition, the confidentiality requirements of the enterprise data or the cooperative relationship between enterprises may change. Enterprises need to update their access control permissions for their APTD and synchronize the relevant information on the blockchain. To this end, we introduce the chameleon hash function designed by Chen et al. [54], which can provide the ability to edit blocks. To improve the efficiency of block editing, we split the trapdoor key of the chameleon hash and design the distributed threshold chameleon hash (DTCH). Each AAE holds a secret key and hash collisions are calculated collaboratively by more than a threshold number of the AAEs. The failure of a few entities will not block the block editing operations. At the same time, group decision-making ensures that the blockchain is not maliciously modified, which can ensure data security.

Moreover, we have designed an accountability mechanism. The accountability mechanism is triggered when block editing fails, and AAEs work together to find malicious entities. Finally, entities can obtain the ciphertext from the system, decrypt it, and then view the APTD.

### 5.2. System Initialization Stage

At this stage, the SA generates system parameters and deploys the redactable blockchain. This scheme adopts the consortium blockchain and entities such as agricultural enterprises need to register with the SA. The SA then provides the entity with an ABE key for data encryption and a hash key for data editing.

The SA selects the security parameter $\lambda_{DTCH}$, records the total number of AAEs as N, and sets the threshold of the DTCH to k. In actual production environments, k can be consistent with the security assumption of consensus algorithms, that is, with the number of active non-malicious AAEs in the system. Running the DTCH.Setup function obtains the system parameter $Param_{DTCH}$.

DTCH.Setup: On inputting security parameter $\lambda_{DTCH}$, the SA selects a GDH group [55] with a generator *g* of order *q*. The SA takes a k−1 degree polynomial $f\left(x\right)=\sum_{i=0}^{k-1}a_{i}x^{i}$, with k−1 coefficients randomly sampled in $Z_{q}^{*}$. This outputs system parameters ${\mathrm{Param}}_{DTCH}=\left\{g,G,H_{0},H_{1},q\right\}$, where $H_{0}:{\left\{0,1\right\}}^{*}\to G^{*},H_{1}:{\left\{0,1\right\}}^{*}\to Z_{q}$.

The entities participating in agricultural product traceability register with the SA. The SA first verifies the identity of the entity and then generates the user secret key $sk_{ABPRE}$ using the VF-CP-ABPRE.KeyGen, based on the system parameter $Param_{ABPRE}$ and the entity’s attribute set S. Next, the SA runs DTCH.KeyGen to generate the secret key of the user.

DTCH.KeyGen: On inputting system parameter $Param_{DTCH}$, the SA randomly selects $x\;\leftarrow \;Z_{q}^{*}$ as the trapdoor key tk, and calculates the public hash key $hk=g^{tk}$. For each AAE $N_{j}$, the SA randomly selects $x_{j}\leftarrow Z_{q}^{*}$, an identity code $\mathrm{UID}\leftarrow Z_{q}^{*}$ [54], and calculates $s_{j}=f\left(x_{j}\right)\;mod\;q$, where j=1,2,…,N.

The SA sends $\left(x_{j},s_{j}\right)$ to $N_{j}$ through a trusted transfer protocol. The entity officially becomes an AAE and participates in the traceability of agricultural products. The AAE has a secret key for signing block editing operations.

The SA sends the public hash key hk and $Param_{DTCH}$ to the redactable blockchain. Subsequently, the SA goes offline and does not participate in the data management process, only providing registration services when new entities are added.

### 5.3. Data Storage Stage

At this stage, agricultural enterprises encrypt and store APTD in the CS and store the summary of APTD on the blockchain. The data are encrypted and uploaded in real-time at the production end. Our solution replaces traditional blockchains with a redactable blockchain, allowing enterprises to use block editing to modify access permissions and update the scope of data sharing.

#### 5.3.1. Data Encryption

**Figure 2:**
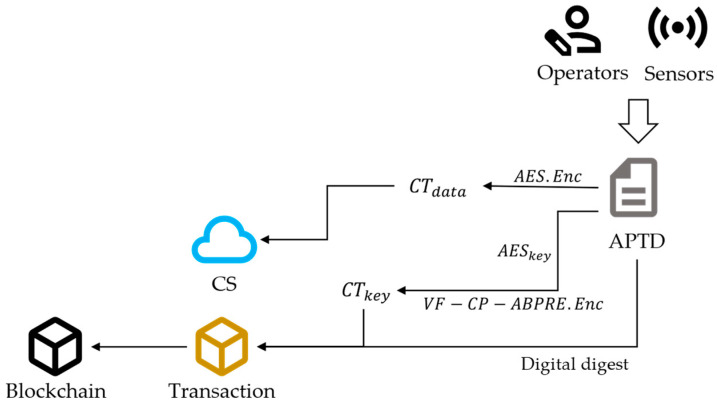
The process of data encryption.

While completing their work, agricultural frontline farmers use mobile terminals and immediately upload APTD to the system. On the other side, IoT devices (such as temperature and humidity sensors) can automatically collect and upload detection APTD. As collectors of APTD, they encrypt the data and upload it to the SA and redactable blockchain, as shown in Figure 2.

In detail, data collectors use symmetric encryption algorithms (such as AES) to encrypt APTD $m_{date}$ and obtain the ciphertext $CT_{data}$. Then, they set the access control policy AP, encrypt the AES key, and obtain $CT_{key}$. At this point, they specify that the data administrator of the affiliated enterprise has access permissions. Subsequently, they use an immutable hash function (such as SHA-256) to create the digital digest DD. The details of this process are shown in Algorithm 1.
**Algorithm 1** Data Encryption**Input:** Original data $m_{data}$ and access policy AP **Output:**
 Ciphertext of data $CT_{data}$, ciphertext of AES key $CT_{key}$, digital digest DD 1: Randomly select key of AES algorithm $key_{AES}$ 2: Calculate $CT_{data}=AES.Enc\left(m_{data},key_{AES}\right)$ 3: Calculate $CT_{key}=VF-CP-ABPRE.Enc\left(key_{AES},AP\right)$ 4: Calculate $DD=SHA-256\left(m_{data}\right)$ 5: **return** $CT_{data},CT_{key},DD$


Data collectors upload $CT_{data}$ to the CS. Finally, they generate a transaction and send it to the miners. This transaction includes $CT_{key}$, AP, and DD. The miners will upload this transaction to the redactable blockchain.

#### 5.3.2. Data Upload

AAEs collect transactions containing APTD summaries, generate blocks, and upload them to the system. By convention, we refer to the entities whose product blocks are uploaded as miners.

**Figure 3:**
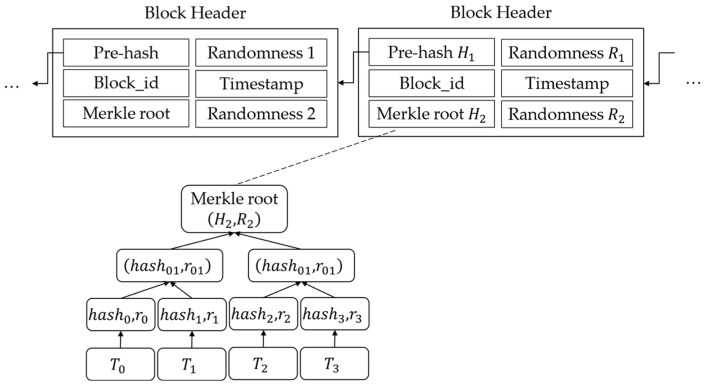
The structure of the block.

After collecting a certain number of transactions, the miner can generate a block and broadcast it, which is composed of two parts, as follows: the block header and the block body. The block header contains the height of the current block, the timestamp of the block when it exits, and the hash value and randomness of the previous block header, as well as Merkle roots and its randomness. The block body stores transactions and we use DTCH.hash to replace the traditional collision-resistant hash in the construction process of the Merkle tree. The structure of the block is shown in Figure 3.

DTCH.Hash: On inputting the public hash key hk, an identity code UID, and the original message m, the miner calculates $c=H_{0}\left(UID,hk\right)$, selects a random number $a\leftarrow Z_{q}^{*}$, and computes $r=\left(g^{a},hk^{a}\right)$. The algorithm outputs hash value $hash=g^{a}c^{H_{1}\left(m\right)}$ and randomness r.

After receiving a new block, entities on the network will verify the correctness of the block. Finally, after reaching a consensus among the majority of entities, the transaction is stored on the blockchain.

### 5.4. Data Editing Stage

In this section, we introduce two scenarios for using block editing and provide details of the editing process. In addition, we introduce the accountability mechanism, which can identify malicious entities when errors occur during the editing process.

#### 5.4.1. Submit Editing Requests

There may be errors in the traceability data of agricultural products that need to be modified and the access control permissions of the data may also need to be updated. In these cases, a block edit can be initiated.

Case 1: Original data error. Due to operator errors or sensor malfunctions, erroneous data may be uploaded to the system. These erroneous data will reduce the credibility of the agricultural product traceability and also affect the accuracy of traceability queries. Therefore, it is necessary to correct these data.

At this point, the provider of the original data (DP) replaces the incorrect data $m_{data}$ with the correct one $m_{data}^{\prime }$. The data $m_{data}$ are encrypted as $CT_{data}$ and stored in the CS. If the DP wants to update the data, they need to re-encrypt the new data $m_{data}^{\prime }$ in the same way. The DP sets the same access policy as the original data. The DP runs Algorithm 1 again to obtain new ciphertext $CT_{data}^{\prime },CT_{key}^{\prime }$, and digital digest $DD^{\prime }$. The DP sends ciphertext $CT_{data}^{\prime }$ to the CS. The DP initiates a block editing request to replace $\left(CT_{key},DD\right)$ stored on the redactable blockchain with $\left(CT_{key}^{\prime },DD^{\prime }\right)$.

Case 2: Change data access permissions. Our scheme utilizes proxy re-encryption technology [52] to allow agricultural enterprises to modify access control permissions. Through this approach, our solution achieves the disclosure and sharing of non-privacy traceability data, which is beneficial for cooperation between agricultural enterprises.

**Figure 4:**
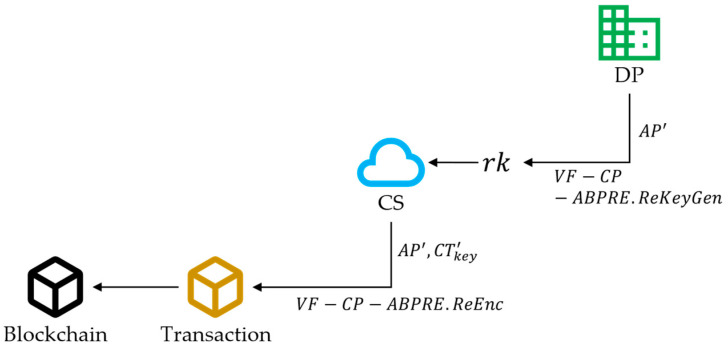
The process of updating data access permissions.

The DP first formulates a new access policy $AP^{\prime }$ and computes the re-encryption key $rk=VF-CP-ABPRE.ReKeyGen\left(sk_{ABPRE},AP^{\prime }\right)$. To reduce the local computing burden, proxy CSs can be used for re-encryption calculations. The CS calculates the re-encrypted ciphertext $CT_{key}^{\prime }=VF-CP-ABPRE.ReEnc\left(CT_{key},rk\right)$. Finally, the DP proposes a block edit, uploads the new ciphertext $CT_{key}^{\prime }$, and updates the access policy to $AP^{\prime }$. In addition, the DP can claim the re-encryption’s correctness by calling the VF-CP-ABPRE.Claim algorithm. This process is shown in Figure 4.

The initiator of the block editing request creates a transaction that modifies the block’s plan and then broadcasts the transaction. Miners will package this transaction to the blockchain. AAEs will evaluate the rationality of the editing plan.

#### 5.4.2. Editing Process

**Figure 5:**
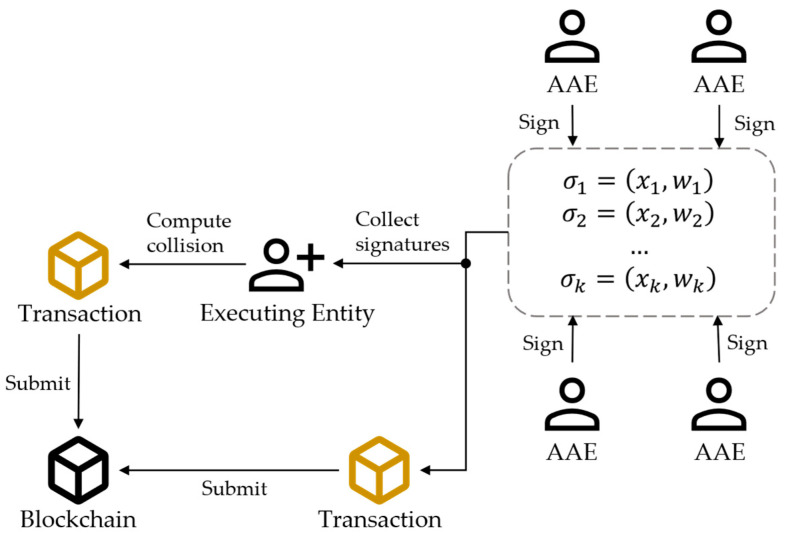
The process of block editing.

The general process of block editing is shown in Figure 5, which can be summarized into three main steps:

Step 1: Issuing signatures. After receiving the editing transaction proposal, each AAE first verifies the rationality of the editing operation. If the AAE does not agree to the editing request, the current editing request will be ignored. Otherwise, the AAE $N_{j}$ generates signature $\sigma_{j}=DTCH.Sign\left(x_{j},s_{j},m,m^{\prime }\right)$ and submits it to other AAEs and the blockchain. This signature will serve as evidence for future accountability. The calculation process of the hash signature is as follows:

DTCH.Sign: On inputting a secret key $\left(x_{j},s_{j}\right)$, an old message m and a new message $m^{\prime }$, $N_{j}$ calculates $\sigma_{j}=\left(x_{j},w_{j}\right)=\left(x_{j},c^{s_{j}\left(H_{1}\left(m\right)-H_{1}\left(m^{\prime }\right)\right)}\right)$.

Step 2: Calculating collisions. The executing entity collects at least k signatures from other AAEs or the blockchain and then runs DTCH.Forge to calculate new randomness $r^{\prime }$. The executing entity broadcasts a submission message with $r^{\prime }$ onto the blockchain. If the executing entity does not collect enough signatures, then this proposal is invalid. The detail of Forge is as follows:

DTCH.Forge: On inputting signatures $\left(\sigma_{1},\sigma_{2},\ldots ,\sigma_{k}\right)$, m, randomness r, and $m^{\prime }$, the executing entity computes $\prod_{j=0}^{k-1}\left({w_{j}}^{\prod_{\begin{array}{c} m=0\\ m\neq j \end{array}}^{k-1}\frac{x_{m}}{x_{m}-x_{j}}}\right)$, $g^{a^{\prime }}=g^{a}\cdot c^{H_{1}\left(m\right)-H_{1}\left(m^{\prime }\right)}$, $hk^{a^{\prime }}=hk^{a}\cdot c^{\sum_{j=0}^{k-1}s_{j}\prod_{\begin{array}{c} m=0\\ m\neq j \end{array}}^{k-1}\frac{x_{m}}{x_{m}-x_{j}}\left(H_{1}\left(m\right)-H_{1}\left(m^{\prime }\right)\right)}$, and $r^{\prime }=\left(g^{a^{\prime }},hk^{a^{\prime }}\right)$. If $g^{a^{\prime }}c^{H_{1}\left(m^{\prime }\right)}=g^{a}c^{H_{1}\left(m\right)}$ and $\langle g,hk,g^{a^{\prime }},hk^{a^{\prime }}\rangle$ are a valid Diffie–Hallman tuple [55], the hash collision is correct; otherwise, it is incorrect.

Step 3: Modification. When AAEs receive a submission message containing the new randomness $r^{\prime }$, they verify the validity of the new hash value. If the verification is successful, AAEs modify the block according to the proposed content $m^{\prime }$ and update the corresponding randomness $r^{\prime }$.

After all the AAEs complete the modifications, the entire blockchain network reaches a consensus on the rewrite operation.

#### 5.4.3. Accountability Mechanism

AAEs may be attacked or engage in malicious behavior for their benefit. On this basis, we further analyze how to identify the perpetrators and deal with various malicious behaviors. These malicious behaviors include (1) malicious entities publishing incorrect signatures in step 1 of the editing process; (2) malicious entities not providing timely signatures in step 1 of the editing process, or maliciously denying editing proposals without providing signatures; and (3) malicious executing entities not strictly modifying blocks according to the content of the editing request.

For case (1), the executing entity which ran DTCH.Forge failed in step 2 of the editing process. Therefore, the executing entity could not obtain a valid hash collision, which indicates the presence of an incorrect signature $\sigma^{\prime }\neq \sigma$. At this point, all AAEs collect signatures and generate a set $L_{1}=\left\{\sigma_{1},\sigma_{2},\ldots ,\sigma_{n}\right\}$. Then, AAEs run Algorithm 2 and obtain a set $L_{2}$ containing all the incorrect signatures. AAEs can find these signatures on the blockchain and identify the corresponding malicious entities that publish these incorrect signatures.
**Algorithm 2** Incorrect Signatures Detection**Input:** A set of signatures $L_{1}=\left\{\sigma_{1},\sigma_{2},\ldots ,\sigma_{n}\right\}$, $\left(hash,m,r,c\right)$, $m^{\prime }$ **Output:** A set of incorrect signatures $L_{3}$ 1: **repeat** 2:  Randomly select k signatures from $\left\{\sigma_{1},\sigma_{2},\ldots ,\sigma_{n}\right\}$ and form a set $\left\{\sigma_{1}^{*},\sigma_{2}^{*},\ldots ,\sigma_{k}^{*}\right\}$ 3:  $r^{\prime }=\left(g^{a^{\prime }},hk^{a^{\prime }}\right)\leftarrow DTCH.Forge\left(\left\{\sigma_{1}^{*},\sigma_{2}^{*},\ldots ,\sigma_{k}^{*}\right\},m,m^{\prime },r\right)$ 4:  $hash^{\prime }\leftarrow g^{a^{\prime }}c^{H_{1}\left(m^{\prime }\right)}$ 5: **until** $\langle g,hk,g^{a^{\prime }},hk^{a^{\prime }}\rangle$ is a valid Diffie–Hallman tuple and $hash=hash^{\prime }$ 6: $L_{2}\leftarrow \left\{\sigma_{1}^{+},\sigma_{2}^{+},\ldots ,\sigma_{k}^{+}\right\}=\left\{\sigma_{1}^{*},\sigma_{2}^{*},\ldots ,\sigma_{k}^{*}\right\}$ 7: $L_{4}\leftarrow L_{1}-L_{2}=\left\{\sigma_{k+1}^{\prime },\sigma_{k+2}^{\prime },\ldots ,\sigma_{n}^{\prime }\right\}$ 8: $L_{2}\leftarrow \emptyset$ 9: **for** $\sigma_{t}^{\prime }$ in $L_{4}$
**do** 10:  $r^{\prime }=\left(g^{a^{\prime }},hk^{a^{\prime }}\right)\leftarrow DTCH.Forge\left(\left\{\sigma_{1}^{+},\sigma_{2}^{+},\ldots ,\sigma_{k-1}^{+},\sigma_{t}^{\prime }\right\},m,m^{\prime },r\right)$ 11:  $hash^{\prime }\leftarrow g^{a^{\prime }}c^{H_{1}\left(m^{\prime }\right)}$ 12:  **if** $\langle g,hk,g^{a^{\prime }},hk^{a^{\prime }}\rangle$ is not a valid Diffie–Hallman tuple or $hash\neq hash^{\prime }$
**then** 13:   add $\sigma_{t}^{\prime }$ into $L_{2}$ 14:  **end if** 15: **end for** 16: **return** $L_{3}$


For case (2), our scheme adopts the distributed threshold chameleon hash function. Editing blocks require approval from AAEs that exceed the threshold but do not require all entities to participate. This means that if an AAE is offline or maliciously disagrees with editing, it will not interrupt the DTCH.Forge operation; hash collisions can still be found normally. We will label AAEs that do not participate in the operation multiple times as malicious entities.

For case (3), the results submitted by the executing entity will be verified by all AAEs. If the executing entity is unable to modify the block information as requested, other AAEs will reject the modification, mark the entity as a malicious entity, and replace the executing entity. To protect the security of the system, malicious entities cannot be selected as the executing entity.

Through the accountability mechanism, we ensure that the block editing operation can be completed, and we can identify the malicious entities. The SA will extract the registration information of the malicious entity and submit it to the agricultural regulatory department.

### 5.5. Data Query Stage

Consumers hope to view the traceability information of agricultural products after purchasing them, such as the planting date, fertilization information, receipt date, transportation time, etc. Consumers can access APTD in our system or entrust data service providers.

Agricultural regulatory authorities will also check the traceability data of agricultural products. For example, regulatory authorities may examine the sources of agricultural product seeds and management during the seedling cultivation process to ensure the quality and purity of the seeds. Regulatory authorities may view information such as sales channels, sales dates, and sales locations, which helps them understand the circulation path of agricultural products and promptly identify potential food safety issues. In summary, regulatory authorities ensure that all aspects of the agricultural product production process comply with national and local regulations and prevent potential violations.

**Figure 6:**
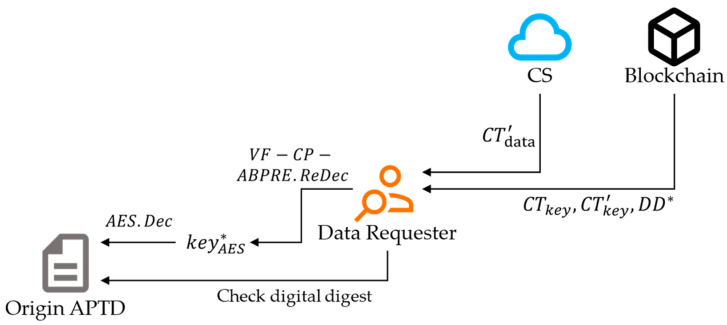
The process of data query.

To obtain APTD, the data requester first searches on the blockchain. The data requester finds a transaction that matches the data they want, and then they obtain the ciphertext $\langle CT_{key},CT_{key}^{\prime }\rangle$ and digital digest DD from the transaction. Additionally, if the access permissions of the data have not been changed, the ciphertext will be $CT_{key}$. Next, the data requester retrieves the ciphertext $CT_{data}$ from the CS. Finally, the data requester calls Algorithm 3 to decrypt the ciphertext and obtain the original APTD. This process is shown in Figure 6.
**Algorithm 3** Data Decryption**Input:** Ciphertext $\langle {\mathrm{CT}}_{\mathrm{key}},{\mathrm{CT}}_{\mathrm{key}}^{\prime }\rangle$ or ${\mathrm{CT}}_{\mathrm{key}}$, access policy *AP* Ciphertext of data *CT_data_* Digital digest *DD* Users’ secret key *sk_ABPRE_* **Output:** Agricultural product traceability data *m_data_* 1: **if**
*sk_ABPRE_* satisfies the access structure *AP* 2:  **if**
${\mathrm{CT}}_{\mathrm{key}}^{\prime }$ is provided **then** 3:    $kde\leftarrow VF-CP-ABPRE.ReDec\left({\mathrm{sk}}_{ABPRE},{\mathrm{CT}}_{key},{\mathrm{CT}}_{key}^{\prime }\right)$ 4:  **else** 5:    $kde\leftarrow VF-CP-ABPRE.OriginDec\left({\mathrm{sk}}_{ABPRE},{\mathrm{CT}}_{key}\right)$ 6: **end if** 7: $mde\leftarrow AES.Dec\left(CT_{data},kde\right)$ 8: **if**
$DD=SHA-256\left(mde\right)$
**then** 9:  return $m_{data}\leftarrow mde$ 10: **else return** ⊥

## 6. Security Analysis

In this section, we analyze the correctness, collision resistance, fault tolerance, and accountability of blockchain editing operations, and then we analyze the privacy of the APTD.

Correctness: For the given $\left(m,r\right)$ and $m^{\prime }$, if the calculating collisions step is successfully executed, a valid $r^{\prime }=\left(g^{a^{\prime }},hk^{a^{\prime }}\right)$ will be obtained, so that $\left(m,r\right)$ and $\left(m^{\prime },r^{\prime }\right)$ can obtain the same hash value. For any $\left(m,r\right)$ and $\left(m^{\prime },r^{\prime }\right)$ pair, there is $hash=g^{a^{\prime }}c^{H_{1}\left(m^{\prime }\right)}=g^{a}\cdot c^{H_{1}\left(m\right)-H_{1}\left(m^{\prime }\right)}c^{H_{1}\left(m^{\prime }\right)}=g^{a}\cdot c^{H_{1}\left(m\right)}c^{H_{1}\left(m^{\prime }\right)-H_{1}\left(m^{\prime }\right)}=g^{a}\cdot c^{H_{1}\left(m\right)}$.

Collision resistance: Assuming that the CDH problem in G is not cracked, DTCH is collision resistant and individuals without the trapdoor key cannot effectively find hash collisions. In the opposite case, it is assumed that the adversary can use a polynomial time algorithm with a non-negligible probability to find hash collisions. Given $\left(m,r\right)$ and $\left(m^{\prime },r^{\prime }\right)$, such that $g^{a}c^{H_{1}\left(m\right)}=g^{a^{\prime }}c^{H_{1}\left(m^{\prime }\right)}$, is equivalent to $Hash\left(m,r,c\right)=Hash\left(m^{\prime },r^{\prime },c\right)$. At this point, the adversary can calculate cx=hka′hkaH1m−H1m′−1 as a solution to the CDH problem on group G. But this means that the adversary can solve the CDH problem on group G, which is not achievable, so the DTCH scheme is collision resistant.

Fault tolerance: Our scheme does not have single-point-of-failure or centralization issues. When the number of non-malicious AAEs is higher than the security threshold, the system can operate normally and the malicious behavior of individual AAEs will not affect the stability of the system. Our scheme adopts the distributed threshold chameleon hash function to implement the redactable blockchain. Editing a block requires approval from AAEs exceeding the threshold number, but it does not require the participation of all AAEs. This means that if an AAE is offline or maliciously disagrees with the edit, it will not affect the efficiency of the DTCH.Forge algorithm, and legitimate editing operations will not be delayed. Collision resistance ensures that malicious AAEs cannot forge fake chameleon hashes. The accountability mechanism can identify malicious AAEs, which regulatory authorities will review and punish.

Accountability: Assuming that the consensus algorithm is safe and effective, valid transactions and editing operations are correctly recorded on the blockchain, providing evidence for accountability. The hash values of transactions are paired in sequence to form a Merkle tree through the hash function. The root of the Merkle tree is stored in the block header. The block header hash value of each block contains the header hash value of the previous block, forming a chain structure. This ensures that each block relies on information from the previous block, forming a chain that cannot be tampered with. Because it is collision-resistant, these structures ensure that transactions are stored securely. The security of the consensus algorithms depends on the consensus algorithm itself chosen by the system, which will not be further elaborated on here.

Privacy protection: Malicious entities cannot decrypt and view APTD. If a malicious entity wants to view data that does not meet its permissions, it must obtain the keys from other entities. To maintain the confidentiality of their data, non-malicious entities will not give out their keys. On the other hand, malicious entities cannot crack the AES and VF-CP-ABPRE algorithms, so they cannot obtain APTD.

## 7. Implementation and Evaluation

In this section, we first test and analyze the performance of data uploading and data editing in our scheme. Then, we test the operating efficiency of the accountability mechanism in the presence of malicious entities. Finally, we analyze the costs of implementing our scheme.

### 7.1. Experiment Setup

We implemented our scheme using the Python 2.8 and Charm framework [56]. We deployed all nodes on servers with an Intel i5-10400F 6-core 2.9GHz CPU, with 4GB of memory, and a 64-bit Linux operating system. We used an MNT224 curve. The consensus algorithm we used is the Practical Byzantine Fault Tolerance (PBFT) algorithm [57] because it is a classic consensus algorithm in the consortium blockchain. Additionally, any secure and efficient consensus algorithm can work. Referring to the security assumption of PBFT, we set the default threshold to k=2n/3. We then tested the time cost of the main steps in the scheme. Our experiment was run 500 times and we took the average value.

### 7.2. Uploading Cost

To store data in the system, the data provider needs to encrypt the original data, encrypt the AES key, and package it into a transaction. Next, the data provider sends the transaction to the miners. After collecting a sufficient number of transactions, a miner packages these transactions into a block. Specifically, the miner calls the DTCH.Hash to calculate the hash value of each node in the Merkle tree until the Merkle root is calculated and then generates the block header to form a complete block. The experimental setup packs different numbers of transactions into a block. At the same time, we compare our scheme with ASCS [22] and PCHBA [33] to compare the efficiency of generating blocks.

**Figure 7:**
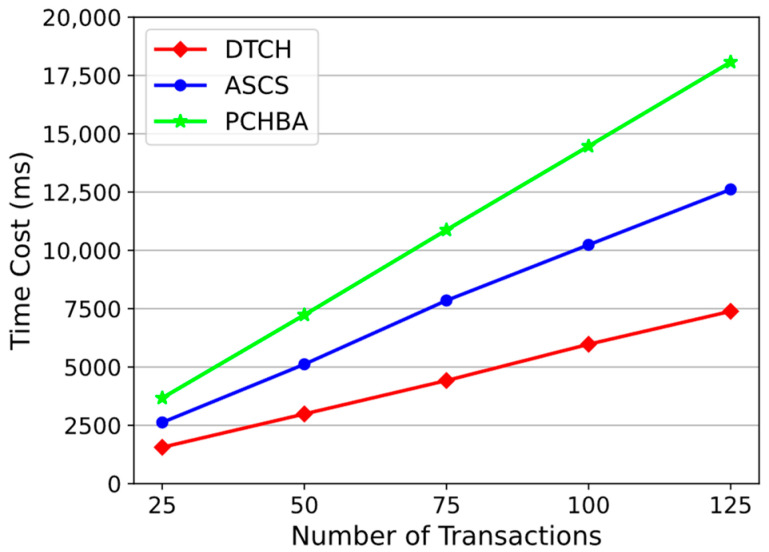
Time cost of storing APTD.

As shown in Figure 7, the time cost of our scheme increases linearly with the number of transactions. Moreover, our scheme has the fastest block generation speed. When 125 transactions are stored in each block, the total time overhead of PCHBA, ASCS, and DTCH (our scheme) are 18,073.77 ms, 12,614.86 ms, and 7388.6 ms, respectively. Our scheme is 42% faster than ASCS and 59% faster than PCHBA. The entire process consists of two steps, as follows: generating transactions and packing blocks. Table 3 shows the specific time overheads of these two steps, as well as both the time to generate transactions and the time to pack blocks, grow with the number of transactions. Our scheme takes 6904.53 ms to generate 125 transactions, accounting for 93.45% of the total time (7388.6 ms); packing blocks takes 484.07 ms, accounting for 6.55%. It can be seen that the vast majority of the time cost is in generating transactions. The packing time cost of ASCS is 487.82 ms, which is similar to that of our scheme, but the time cost of generating transactions is more, 12,127.04 ms. The ASCS scheme uses the ASCS.SS signature algorithm when packaging transactions. It needs to split the original data into multiple parts, calculate hash values, and sign them separately. This means that the TCH algorithm and BLS signature algorithm need to be run multiple times, which takes up a lot of time. The PCHBA scheme is the slowest in packaging transactions, it takes 18,072.18 ms to package 125 transactions, due to its use of additional cryptographic primitives in its hashing algorithm, including hierarchy identity-based encryption and Schnorr signature algorithms. It has the fastest block generation speed, only 1.59 ms, because it uses SHA-256 to build a Merkle tree but not a chameleon hash function. This results in this scheme only providing transaction-level editing, but not the Merkle tree or block-level editing.

### 7.3. Editing Cost

The block-editing stage includes four parts, as follows: proposal, signature, collision calculation, and submission. Among them, the proposal operation is executed off-chain. The efficiency of the submission step is related to the system’s consensus algorithm. Analyzing the efficiency of the consensus algorithm is beyond the scope of this article.

Therefore, what affects the efficiency of the editing operation in our scheme is the signature step and the collision calculation step. The running process of this experiment will include these two steps. AAEs receive the edit request and use their secret key to run DTCH.Sign to obtain the signature and upload it to the blockchain. After the executing entity collects a sufficient number of signatures, it runs DTCH.Forge to calculate the hash collision.

**Figure 8:**
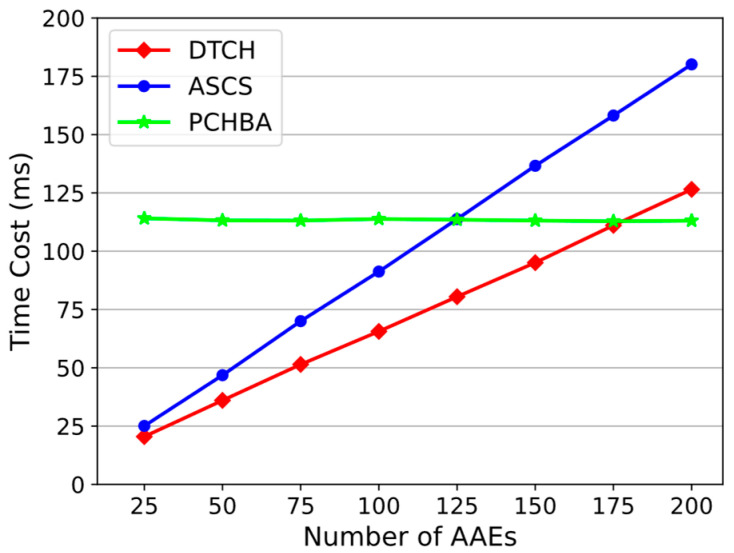
Time cost of editing blocks.

For the same block edit, the experiment sets come to a total of different AAE numbers, increasing from 25 to 200 with a step size of 25. As shown in Figure 8, the time overhead of ASCS and DTCH (our scheme) increases linearly with the number of AAEs, and the difference is getting bigger. Overall, our scheme has less overhead than ASCS. When the number of AAEs is 200, the time overhead of our scheme is 126.5 ms and ASCS is 180.12 ms. The ASCS scheme connects sensors into a ring. The signature of the previous sensor is passed to the subsequent sensor and the hash collision is obtained after the calculation of the last sensor is completed. The signature operations of each sensor are executed serially, which slows down the execution efficiency of the collision search. Our solution is faster because, during the signature phase, each AAE performs DTCH.Sign locally at the same time, which is equivalent to parallel operations and saves the time of waiting for each other. The speed of the PCHBA scheme is constant, with an average of 113.36 ms, independent of the number of AAEs. Because there is no distributed key management, a single AAE with sufficient access rights can complete block editing. When the number of AAEs is small, PCHBA has greater time overhead than the other two schemes, while when the number of AAEs is large, PCHBA is more advantageous in terms of time overhead. Specifically, PCHBA outperforms ASCS when the number of AAEs is greater than 125. When the number of AAEs is approximately greater than 177, the time overhead of PCHBA will be less than our scheme. Overall, when the number of AAEs is small, our scheme outperforms the other two schemes in terms of editing overhead.

### 7.4. Performance Evaluation of the Accountability Mechanism and Discussion

There may be malicious AAEs. To evaluate the stability of the scheme, we test the operating efficiency of the accountability mechanism under different numbers of malicious AAEs and different total numbers of AAEs. Malicious AAEs may upload incorrect signatures in the issue signatures step. These incorrect signatures will interrupt the editing operation and trigger the accountability mechanism. We set the total number of AAEs to 40, 60, 80, 100, and 120 and the number of incorrect signatures to 1, 2, and 3 to count the time overhead of the accountability mechanism.

**Figure 9:**
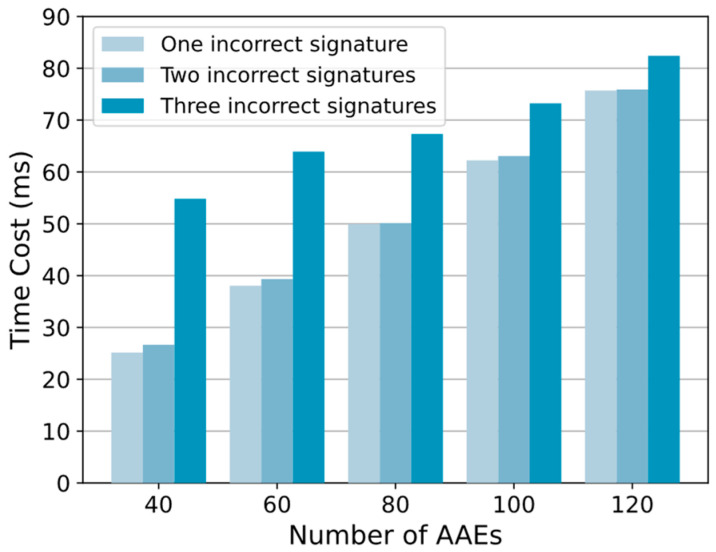
Time cost of accountability mechanisms with different malicious entities.

As shown in Figure 9, the number of incorrect signatures in each group increases sequentially from left to right. The running time increases with the total number of AAEs and also with the number of incorrect signatures. For example, when the number of AAEs is 120 and the number of erroneous signatures is 1, 2, and 3, the time overhead of the accountability mechanism is 75.71 ms, 75.92 ms, and 82.42 ms, respectively. When the number of erroneous signatures is 3 and the number of AAEs is 40, 60, 80, 100, and 120, the time overhead of the accountability mechanism is 54.83 ms, 63.93 ms, 67.33 ms, 73.24 ms, and 82.42 ms. Every time a wrong signature is found, the corresponding malicious AAE will be marked, so the number of such malicious AAEs in the network is small and the accountability mechanism will not be triggered frequently.

In addition, the proportion of non-malicious AAEs in different scenarios is different, and the security assumptions of the consensus algorithm may also be different, which means that the number of potentially malicious AAEs in the system is different. When it is assumed that the number of malicious AAEs is small and the number of non-malicious AAEs is large, the DTCH threshold k can be lowered in exchange for a faster accountability calculation speed.

**Figure 10:**
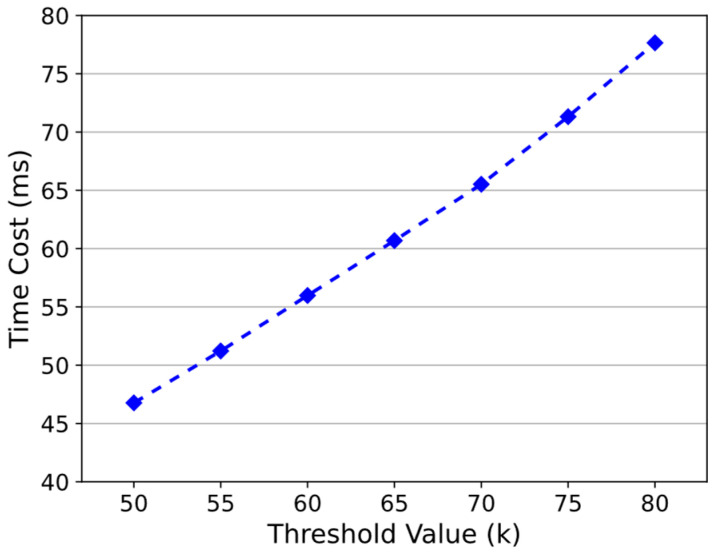
Time cost of accountability mechanisms with different threshold values.

The experiment sets the number of incorrect signatures to 2, the total number of AAEs to n=100, the step size to 5, and lets k be 50, 55, 60, 65, 70, 75, and 80, respectively. As shown in Figure 10, time cost grows with k. Specifically, the results were 46.76 ms, 51.22 ms, 55.98 ms, 60.69 ms, 65.52 ms, 71.32 ms, and 77.65 ms. As k decreases, the core operation of Algorithm 2, DTCH.Forge, will perform fewer multiplication operations, which reduces the computational overhead. Therefore, in practical applications, the threshold k can be appropriately set according to actual needs.

### 7.5. Implementation Cost Analysis

In this subsection, we analyze the costs of implementing our scheme, including several components, as follows:

Blockchain nodes: AAEs need to set up and maintain blockchain nodes, which are computers or servers that participate in the blockchain network. The nodes store copies of the blockchain and verify transactions. The time overhead of the core computing process has been tested in the experimental part and the overhead growth is stable and meets the application requirements.

Storage cost: Cloud service providers typically charge based on the amount of storage space utilized and charge for data transfers to and from their storage services. Cloud providers may offer different service levels with different guarantees for performance, availability, and reliability. Higher service levels typically mean higher costs but offer better performance and availability guarantees. Implementing redundancy and backup solutions can ensure data availability and persistence, which can incur additional costs. With limited budgets, we recommend that AAEs co-maintain the IPFS (InterPlanetary File System) in place of third-party cloud databases to reduce expenses. The IPFS is a decentralized file system where files are stored and distributed across multiple nodes in a network, allowing files to be fetched directly from other nodes, which results in efficient transfer and high stability.

Network Cost: Payment for network bandwidth to support communication between blockchain nodes, data transfer, and transaction propagation in the blockchain network. The cost of network bandwidth may vary depending on factors such as the amount of data transferred, network speed, and service provider pricing. The blockchain in this scenario only stores information such as APTD digests and not large data, so the communication overhead of the blockchain network will not be too high.

Integration and interoperability of blockchain systems with existing data sources and platforms: To achieve traceability of agricultural products, blockchain systems need to collect and store data from a variety of sources, such as sensors, IoT devices, RFID tags, QR codes, and databases. These data sources may have different formats, standards, and protocols and require data conversion.

Maintenance of blockchain systems: blockchain systems require regular maintenance and updates to ensure their functionality, security, and reliability. This may involve hardware and software upgrades, bug fixes, backups and restores, and audits.

While upfront costs may be higher compared to traditional methods, the benefits of enhanced transparency, invariance, and trustworthiness offered by blockchain technology can provide a long-term value and mitigate the risks associated with traditional traceability systems.

## 8. Conclusions

Agricultural product traceability requires data to be shared whilst protecting data privacy and ensuring that data are not tampered with. Blockchains are known for their immutability and openness and they are often used for storing data. However, the blockchain has scalability issues and cannot delete or update data. In this paper, we proposed an agricultural product traceability data management scheme based on a redactable blockchain. We used proxy re-encryption attribute-based encryption algorithms to encrypt data and protect enterprise privacy. Enterprises can use the chameleon hash function to fix erroneous data and update access control permissions. Furthermore, we improved the distributed threshold chameleon hash function to distribute block editing permissions among authorized entities; the disconnection of a few entities does not affect the normal progress of data updating. We established an accountability mechanism to identify malicious entities. Finally, our experiment proved that the proposed scheme has advantages in terms of speed compared to existing methods and can meet the requirements of practical applications. In the future, we will further optimize the editing scheme to improve its data storage and query speed capabilities. We will also add incentive mechanisms to encourage more companies to participate in data sharing.

## Figures and Tables

**Table 1:** Comparison of different data management schemes based on redactable blockchains.

Author	Distributed Editing Privilege	Key Protection	Modifier	Accountability
Ateniese et al. [18]	No	No	(t,n)-threshold	No
Deuber et al. [31]	Yes	/	All miners	Yes
Derler et al. [32]	No	Yes	Authorized entity	No
Huang et al. [22]	Yes	Yes	(n,n)-threshold	No
Tian et al. [33]	No	Yes	Authorized entity	Yes
Duan et al. [45]	No	Yes	Authorized entity	Yes
Ours	Yes	Yes	(t,n)-threshold	Yes

**Table 2:** Notation description.

Notion	Description
SA	System administrator
AAE	Authorized agricultural entity
CS	Cloud server
APTD	Agricultural product traceability data
$\lambda_{DTCH}$	Security parameters of chameleon hash
$\lambda_{ABPRE}$	Security parameters of VF-CP-ABPRE
$Param_{DTCH}$	System parameters of chameleon hash
$Param_{ABPRE}$	System parameters of VF-CP-ABPRE
hk	Public key of chameleon hash
tk	Trapdoor key of chameleon hash
$Z_{q}^{*}$	The integer modulus q multiplication group
$H_{0},H_{1}$	Collision-resistant hash functions
G	Gap Diffie–Hellman (GDP) group
$\left(x,s\right)$	Chameleon hash secret key of AN
k	Threshold parameter of chameleon hash
UID	Identity code of AN
σ	Chameleon hash signature of AN
$sk_{ABPRE}$	VF-CP-ABPRE secret key of AN
$CT_{data}$	Ciphertext of APTD
AP	Access policy of APTD
S	Attribute set of AAE
$sk_{AES}$	Secret key of AES
$CT_{key}$	Ciphertext of AES key
DD	Digital digest of APTD
rk	Re-encryption key of VF-CP-ABPRE

**Table 3:** Time cost (ms) of generating a block with different number of transactions.

Number of Transactions	DTCH		ASCS		PCHBA	
	Generate Transactions	Package Block	Generate Transactions	Package Block	Generate Transactions	Package Block
25	1464.76	93.42	2529.41	93.72	3670.52	0.44
50	2793.44	192.39	4925.18	191.95	7233.42	0.56
75	4127.84	291.52	7560.19	292.23	10,873.72	0.84
100	5586.88	388.01	9850.36	387.61	14,466.85	1.11
125	6904.53	484.07	12,127.04	487.82	18,072.18	1.59

## Data Availability

Data are contained within the article.
